# Newly identified HMO-2011-type phages reveal genomic diversity and biogeographic distributions of this marine viral group

**DOI:** 10.1038/s41396-021-01183-7

**Published:** 2022-01-12

**Authors:** Fang Qin, Sen Du, Zefeng Zhang, Hanqi Ying, Ying Wu, Guiyuan Zhao, Mingyu Yang, Yanlin Zhao

**Affiliations:** 1 Fujian Provincial Key Laboratory of Agroecological Processing and Safety Monitoring, College of Life Sciences, Fujian Agriculture and Forestry University, Fuzhou, China; 2 Key Laboratory of Marine Biotechnology of Fujian Province, Institute of Oceanology, Fujian Agriculture and Forestry University, Fuzhou, China

**Keywords:** Bacteriophages, Biodiversity, Microbial ecology

## Abstract

Viruses play critical roles in influencing biogeochemical cycles and adjusting host mortality, population structure, physiology, and evolution in the ocean. Marine viral communities are composed of numerous genetically distinct subfamily/genus-level viral groups. Among currently identified viral groups, the HMO-2011-type group is known to be dominant and broadly distributed. However, only four HMO-2011-type cultivated representatives that infect marine SAR116 and *Roseobacter* strains have been reported to date, and the genetic diversity, potential hosts, and ecology of this group remain poorly elucidated. Here, we present the genomes of seven HMO-2011-type phages that were isolated using four *Roseobacter* strains and one SAR11 strain, as well as additional 207 HMO-2011-type metagenomic viral genomes (MVGs) identified from various marine viromes. Phylogenomic and shared-gene analyses revealed that the HMO-2011-type group is a subfamily-level group comprising at least 10 discernible genus-level subgroups. Moreover, >2000 HMO-2011-type DNA polymerase sequences were identified, and the DNA polymerase phylogeny also revealed that the HMO-2011-type group contains diverse subgroups and is globally distributed. Metagenomic read-mapping results further showed that most HMO-2011-type phages are prevalent in global oceans and display distinct geographic distributions, with the distribution of most HMO-2011-type phages being associated with temperature. Lastly, we found that members in subgroup IX, represented by pelagiphage HTVC033P, were among the most abundant HMO-2011-type phages, which implies that SAR11 bacteria are crucial hosts for this viral group. In summary, our findings substantially expand current knowledge regarding the phylogenetic diversity, evolution, and distribution of HMO-2011-type phages, highlighting HMO-2011-type phages as major ecological agents that can infect certain key bacterial groups.

## Introduction

Viruses in the marine environment are extremely abundant and diverse, and play critical roles in nutrient cycling and microbial community function and structure [1–5]. Viruses contribute substantially to the mortality of marine microorganisms, and thus play a key role in shaping the structure and function of microbial communities, and this, in turn, affects marine biogeochemical cycles [1–4]. Moreover, viruses can drive microbial evolution and diversification by serving as a selective pressure and mediating lateral gene transfer [1–5]. Although the vital functions of viruses in marine ecosystems are now widely recognized, we have just begun to explore their genetic diversity and ecological functions and the tremendous biodiversity of marine viral communities are not well understood yet. Over the past decade, culture-independent metagenomic and single-cell genomic technologies have been increasingly used to characterize marine viral communities [6–15], and with the development of sequencing and assembly method, the number of metagenome-assembled viral genomes has increased dramatically [7, 9, 12, 14–17]. Furthermore, third-generation sequencing have been applied to generate long-read data in some viral metagenomic studies [18, 19]. Metagenomic datasets also represent valuable resources for investigating viral biogeography in marine environments [20–27]. Although these advances have substantially expanded our understanding of the genetic composition of marine viruses, most of the sequences in marine viromes show no homology to known phages and remain uncharacterized; thus metagenomic research currently is limited in enabling experimental identification of phage-host identity and answering questions regarding virus–host interactions in natural viral populations. Therefore, elucidation of the ecological, physiological, and evolutionary role of marine bacteriophages is challenging and requires studies on laboratory virus–host systems.

As compared to the rapid advance in metagenomic studies, advances in phage isolation efforts have been slower and fewer isolated phages are available due to the challenge encountered in the culturing of many bacteria and their phages in the laboratory. Although culture-dependent studies have lagged behind culture-independent metagenomic investigations, several recent phage cultivation studies have enabled the discovery of many important phages that infect ecologically important marine bacteria, with the most notable cases including SAR11 phages (pelagiphages), *Roseobacter* RCA phages, and SAR116 phage [20–22, 26–28]. The bacteria within the SAR11, SAR116 and certain *Roseobacter* lineages are abundant and widespread groups of heterotrophic bacteria that dominate the ocean surface [29–33]. Phages isolated from members of these bacterial groups have been shown to be diverse and abundant in marine environments [20–22, 27]. Moreover, some of the phage isolates were found to match some abundant viral groups identified using metagenomic analysis [27]. In this regard, phage isolates are critical for interpreting metagenomic data and identifying the potential hosts for the metagenomic sequences. Therefore, both culture-dependent and -independent methods are indispensable for investigating marine viruses.

Among the identified viral groups, the HMO-2011-type group is considered one of the most abundant and widespread viral groups [20, 22]. Short-tailed HMO-2011-type phages are members of the *Caudovirales* order, with a double-stranded DNA genome. HMO-2011-type group has four cultivated representatives so far [20, 22]. *Puniceispirillum* phage HMO-2011, which infects marine SAR116 strain IMCC1322, was the first cultivated phage in this group. Metagenomic reads related to HMO-2011 were found to be abundant in several marine viromes [20, 34]. More recently, three additional HMO-2011-type phages (CRP-1, CRP-2, and CRP-3) that infect *Roseobacter* RCA strains were isolated and characterized, and this led to the speculation that RCA phages contribute to the dominance of the HMO-2011-type group [22]. Genomic analyses have revealed that HMO-2011-type phages possess a novel DNA polymerase gene with unique domain architecture [20, 22]. Despite the recent discoveries related to this viral group, the genomic diversity and ecology of this group are poorly understood and whether HMO-2011-type phages can infect a more diverse range of hosts remains to be investigated.

Our main aim in this study was to investigate the genomic diversity and global prevalence of HMO-2011-type phages. We report seven newly isolated HMO-2011-type phages, which were isolated using four *Roseobacter* strains and one SAR11 strain as the hosts. Moreover, we performed metagenomic mining to identify HMO-2011-type metagenomic viral genomes (MVGs). Our genomic and phylogenetic analyses revealed that HMO-2011-type viral group is composed of diverse subgroups, and the results of the metagenomic analysis showed that these HMO-2011-type phages are widely distributed in the world’s oceans and exhibit distinct global distribution patterns.

## Materials and methods

### Host strains and growth conditions

*Roseobacter* strains FZCC0040, FZCC0042, FZCC0012, and FZCC0089 were isolated on May 13, 2017, from the coastal waters of Pingtan Island in China (25°26′N, 119°47′E) by using the dilution-to-extinction method. All *Roseobacter* strains were cultured in natural seawater-based medium supplemented with 1 mM NH_4_Cl, 100 μM KH_2_PO_4_, 1 μM FeCl_3_ and mixed carbon sources [35], and cultured at 23 °C. The SAR11 strain *Pelagibacter* sp. HTCC1062 was kindly provided by Prof. Stephen Giovannoni, Oregon State University. HTCC1062 was cultured in artificial seawater-based ASM1 medium [36] supplemented with 1 mM NH_4_Cl, 100 μM KH_2_PO_4_, 1 μM FeCl_3_, 100 μM pyruvate, 50 μM glycine, and 50 μM methionine. HTCC1062 were grown in the dark without shaking at 17 °C. The concentration of bacterial cells was determined using a Guava EasyCyte flow cytometer (Millipore, Guava Technologies) after staining with SYBR Green I (Invitrogen).

### Phage isolation and purification

The seawater samples used for isolating the bacteriophages were collected from three oceanic sampling stations (Table 1). Samples were filtered using 0.1 μm-pore-size sterile syringe filters and stored in the dark at 4 °C until use. The procedures for phage isolation and purification have been described in detail previously [21, 22, 28]. Briefly, filtered seawater samples were added into the host cultures and cell growth was monitored using a Guava EasyCyte cell counter. When a decrease in cell density was detected, the presence of phage particles was confirmed using epifluorescence microscopy. The phages were purified using the dilution-to-extinction method [21, 22, 37], with the purification procedures being repeated three times to ensure that a single pure line of each phage was obtained. The purity of the isolated phages was verified using whole-genome sequencing.

**Table 1:** General features of seven HMO-2011-type phages sequenced in this study.

Phage	Original host	Source water	Depth	Latitude	Longitude	Collection date	Genome size (bp)	Number of ORFs	%G + C	Accession number
CRP-207	FZCC0040	Pattaya Beach, Thailand	Surface	N 12°56′	E 100°53′	March 2018	54895	76	46.2	MZ892987
CRP-235	FZCC0040	North Sea	6 m	N 53°56′	E 7°48′	March 2019	52729	61	45.7	MZ892989
CRP-603	FZCC0012	Pattaya Beach, Thailand	Surface	N 12°56′	E 100°53′	March 2018	54551	77	43.1	MZ892991
CRP-345	FZCC0042	Pattaya Beach, Thailand	Surface	N 12°56′	E 100°53′	March 2018	54718	81	42.2	MZ892990
CRP-212	FZCC0040	Pattaya Beach, Thailand	Surface	N 12°56′	E 100°53′	March 2018	54748	59	48.6	MZ892988
CRP-738	FZCC0089	Pattaya Beach, Thailand	Surface	N 12°56′	E 100°53′	March 2018	53826	65	45.6	MZ892992
HTVC033P	HTCC1062	Mediterranean Sea	Surface	N 43°42′	E 7°17′	August 2016	53075	84	33.8	MZ892993

### Phage DNA extraction, genome sequencing, and assembly

Phage particles were concentrated from 200 mL of cell lysates as previously described [22]. Briefly, each phage lysate was filtered through 0.1 μm filters and concentrated to 300 μL by using Amicon Ultra Centrifugal Filters (30 kDa, Millipore) and Nanosep centrifugal devices (30 kDa, PALL). Phage genomic DNA was extracted using the formamide treatment, phenol-chloroform extraction method [38] and sequenced on an HiSeq 2500 platform (Illumina) with a paired-end read length of 150 bp. Quality-filtering, trimming and de novo assembly were performed by using CLC Genomic Workbench v11.0.1 (Qiagen, Hilden, Germany) with default settings. The remaining gaps were closed through Sanger sequencing of PCR products covering the gap areas.

### Metagenomic retrieval of HMO-2011-type MVGs

For our analyses, MVGs reconstructed from Global Ocean Viromes (GOV and GOV 2.0) [12, 14], the MedDCM fosmid library [7], Station ALOHA assembly-free virus genomes [19], and the ALOHA 2.0 viromic database [15] were downloaded for analyses. Open reading frames (ORFs) of MVGs were predicted using prodigal [39]. Here, we used three HMO-2011-type hallmark genes, including the DNA polymerase (DNAP), capsid and terminase large subunit (TerL) genes, as baits to retrieve the HMO-2011-type phage genomes. Profile hidden Markov models (HMM) were constructed using DNAP, capsid and TerL protein sequences of HMO-2011-type isolates using hmmbuild with default parameters [40]. The HMM profiles were used to query the downloaded MVGs using hmmsearch program (e-value ≤10^−3^ and score ≥50). Only matches with ≥25% identity and ≥80% alignment length were considered. MVGs that contain all three gene homologs were retained for further analysis. For HMO-2011-type DNAP homologs, the DnaJ domains and two CXXCXGXG motifs were identified by manually checking the sequences [20], and only the MVGs whose DNAP sequences contain a partial DNAJ domain and two CXXCXGXG motifs were considered as HMO-2011-type phages. CheckV was used for completeness and quality estimation of these HMO-2011-type MVGs [41]. MVGs with a genome completeness ≥50% were used for further phylogenomic and comparative genomic analyses.

### Genome annotation and comparative genomic analysis

The GeneMark online server [42] and Prodigal [39] were used to predict ORFs from all HMO-2011-type genomes. Translated ORFs were analyzed and annotated by BLASTP and PSI-BLAST against the NCBI nonredundant and NCBI Refseq databases (e-value ≤10^−3^; ≥25% amino acid identity; ≥50% alignment length). ORFs were searched against the Pfam database with HMMER web server [43, 44] for recognizable conserved PFAM domains. For structure and function prediction, we also used the Conserved Domain Search Service of NCBI [45] and HHpred server [46]. ORFs were assigned putative biological functions according to the function of proteins encoded by homologous genes. tRNAscan-SE was used to identify tRNA genes [47]. OrthoFinder v2.5.2 [48] was used to identify groups of orthologous genes from different HMO-2011-type genomes based on sequence similarity (BLASTP option: e-value ≤10^−3^; ≥25% identity; ≥50% alignment length). Representative HMO-2011-type genomes were compared and visualized using Easyfig v2.2.2 [49].

### Phylogenomic analyses

We conducted phylogenomic analyses to evaluate the evolutionary relationship of HMO-2011-type phages. Five core genes were selected for phylogenomic analysis (genes encoding DNA helicase, DNAP, capsid, portal and TerL). The core genes were aligned using MUSCLE [50] and edited using Gblocks [51]. The alignments were concatenated and a phylogenetic tree was constructed using IQ-TREE v1.6.12 [52] with 1000 bootstrap replicates. The whole-genome phylogenetic tree based on amino acid sequences was also constructed using GL-UVAB workflow [53] with the Dice coefficient under default settings. The taxonomic classification of HMO-2011-type phages at the genus level was performed according to the recommended minimum node depth of 0.0189 and number of representatives equal or above 3. The phylogenetic trees were visualized and annotated using Interactive Tree Of Life (iTOL) v.5 [54].

### Host prediction

The potential hosts of HMO-2011-MVGs were predicted using RaFAH tool with default settings [55]. The training and validating random forest model for RaFAH was built with 4269 host-known phages, including 11 HMO-2011-type phages and 4258 bacteriophage genomes downloaded from the NCBI RefSeq (v208).

### Identification and phylogenetic analysis of HMO-2011-type DNAP sequences

A hidden Markov profile (HMM) made from an alignment of DNAP gene sequences was used to query the downloaded MVGs using hmmsearch program (e-value ≤10^−3^ and score ≥50). The DnaJ domains and two CXXCXGXG motifs were identified by manually checking the sequences. DNAP sequences with ≥80% coverage length and size larger than 540 aa were used for the phylogenetic analysis. GOV 2.0 viral populations were searched using hmmsearch [40] to identify DNAP family A (PF00476) sequences.

The amino acid sequences of all HMO-2011-type DNAP were aligned using MUSCLE [50] and edited using Gblocks [51] for phylogenetic analysis. A maximum-likelihood phylogenetic tree was constructed by using IQ-TREE v1.6.12 [52] with 1000 bootstrap replicates.

### Recruitment of metagenomic reads and statistical analysis

The relative abundance of HMO-2011-type phages in marine viromes was estimated through a viromic read-mapping analysis. Global Oceans Viromes (GOV 2.0) were downloaded for accessing the relative abundance [14]. HMO-2011-type genomes were compared using NUCmer [56]. Genomes sharing ≥95% nucleotide identity across ≥80% of the genome were classified into a single species, and only the longest MVGs within a species were retained for recruitment analysis. Viromic reads were mapped against the nonredundant set of HMO-2011-type genomes by using BLASTN (≥95% nucleotide identity over ≥90% read coverage). The relative abundances of HMO-2011-type phages were normalized by the total recruited nucleotides (kb) per kilobase of genome per gigabase of metagenome (KPKG). HMO-2011-type genomes for which <40% of the genomes were covered by recruited viromic reads in a given viromic dataset were regarded as absent and were assigned a KPKG value of 0 [26]. Heatmap was plotted using R package pheatmap. Linear-regression analysis generated by R was used to test the relationship between environmental parameters and relative abundance of HMO-2011-type phages. Box plots of the pelagiphages KPKG were plotted using R package ggplot2.

## Results and discussion

### General characterization of seven newly isolated HMO-2011-type phages

In this study, we used four *Roseobacter* strains (FZCC0040, FZCC0042, FZCC0012, and FZCC0089) and one SAR11 strain (HTCC1062) to isolate phages. FZCC0040 and FZCC0042 belong to the *Roseobacter* RCA lineage [22], FZCC0012 shares 99.8% 16S rRNA gene identity with *Roseobacter* strain HIMB11 [57], and FZCC0089 belongs to a newly identified *Roseobacter* lineage located close to HIMB11 and SAG-019 lineages (Supplementary Fig. 1).

A total of seven phages were newly isolated and analyzed in this study (Table 1). The complete phage genomes range in size from 52.7 to 54.9 kb, harbor 62 to 84 open reading frames (ORFs), and feature a G + C content ranging from 33.8 to 48.6%. Compared to other HMO-2011-type phages, pelagiphage HTVC033P has a relatively lower G + C content of 33.8%, similar to the G + C content of its host HTCC1062 (29.0%) and of other described pelagiphages [21, 26–28]. The G + C content of other six roseophages ranges from 42.2 to 48.6%, which is also similar to the G + C content of the hosts they infect (44.8 to 54.1%).

**Fig. 1:**
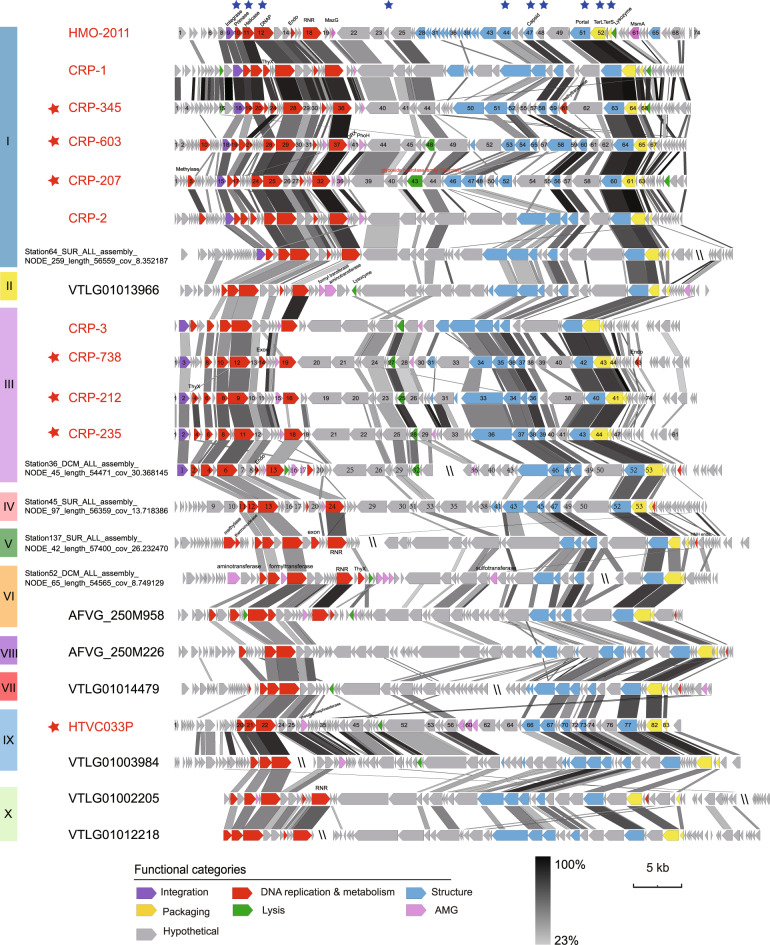
Alignment and comparison of genomes of HMO-2011-type isolates and representative HMO-2011-type MVGs from major subgroups. HMO-2011-type phage isolates are shown in red. Phages isolated in this study are indicated with red asterisks. Predicted open reading frames (ORFs) are represented by arrows, with the left or right arrow points indicating the direction of their transcription. The numbers inside the arrows indicate ORF numbers. ORFs annotated with known functions are marked using distinct colors according to their functions. HMO-2011-type core genes are indicated with blue asterisks. The color of the shading connecting homologous genes indicates the level of amino acid identity between the genes. To clearly present the genomic comparison, several MVGs were rearranged to start from the same gene as in the HMO-2011-type phages. DNAP DNA polymerase, Endo endonuclease, RNR ribonucleoside-triphosphate reductase, PhoH phosphate starvation-inducible protein, MazG MazG nucleotide pyrophosphohydrolase domain protein, ThyX thymidylate synthase, GRX glutaredoxin, TerS terminase small subunit, TerL terminase large subunit.

Despite their distinct host origins, these phage genomes show considerable similarity in terms of gene content and genome architecture (Fig. 1). They all display clear similarity with the previously reported SAR116 phage HMO-2011 [20] and HMO-2011-type RCA phages [22]. Overall, these phages share 19.2 to 79.1% of their genes with previously reported HMO-2011-type phages and all contain homologues of HMO-2011-type DNA replication and metabolism genes, structural genes, and DNA packaging genes. Moreover, their overall genome structure is conserved with that of HMO-2011-type phages. Considering these observations, we tentatively classified these seven phages into the HMO-2011-type group. Of the 11 currently known HMO-2011-type isolates, one infects the SAR116 strain IMCC1322, one infects the SAR11 strain HTCC1062, and the remaining nine all infect *Roseobacter* strains; this suggest that HMO-2011-type phages infect diverse bacterial hosts. HTVC033P is the first pelagiphage identified to belong to the HMO-2011-type viral group. Our study has also increased the number of known types of pelagiphages. To date, pelagiphages belonging to a total of nine distinct viral groups have been isolated and analyzed [21, 26–28].

### Identification and sequence analyses of HMO-2011-type MVGs

To identify HMO-2011-type MVGs, we performed a metagenomic mining and retrieved a total of 207 HMO-2011-type MVGs (≥50% genome completeness) from viromes in the worldwide ocean, from tropical to polar oceans (Supplementary Table 1). These MVGs range in size from 29.2 to 67.9 kb and their G + C content range from 31.3 to 52.4%. In addition, 45 HMO-2011-type MVGs were also identified from some non-marine habitats, suggesting that HMO-2011-type phages are widely distributed worldwide (Supplementary Table 1).

**Fig. 2:**
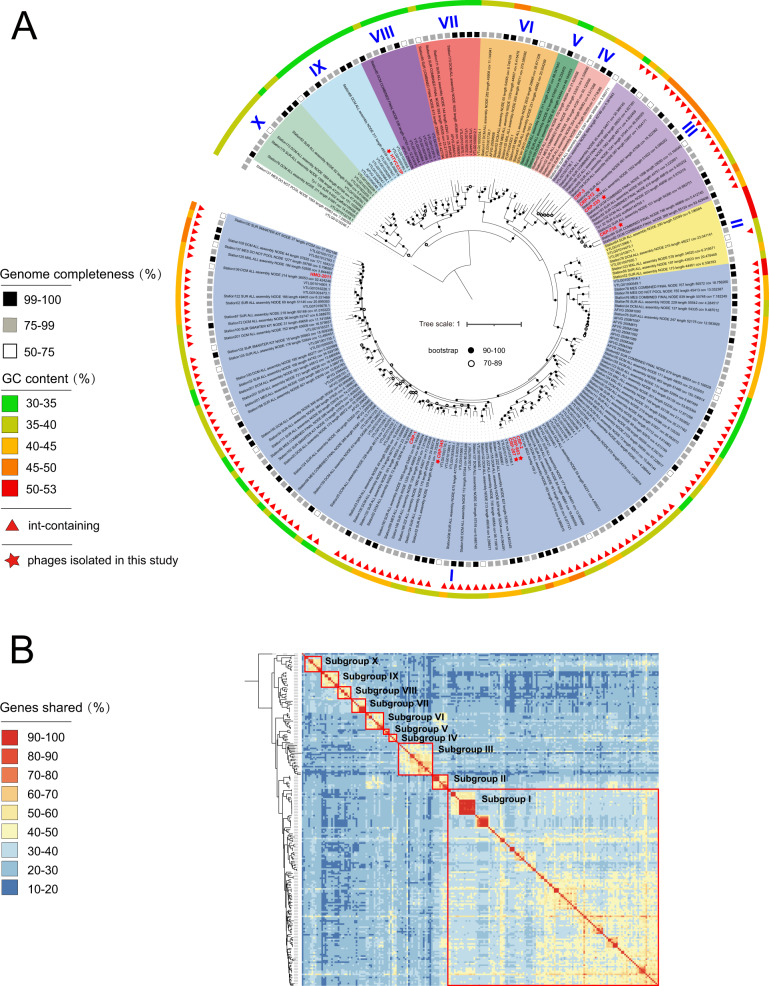
Phylogenomic and shared-gene analyses of HMO-2011-type phages. **A** A maximum-likelihood tree was constructed using concatenated sequences of five hallmark genes. HMO-2011-type phages were grouped into 10 subgroups based on the phylogeny. Shading is used to indicate the subgroups. HMO-2011-type phage isolates are shown in red. Genomes containing an integrase gene are indicated by red triangles. The G + C content and completeness of the genomes are indicated. Scale bar indicates the number of amino acid substitutions per site. **B** Heatmap showing the percentage of shared genes between HMO-2011-type genomes. Phages in the same subgroup are boxed.

Genomic analysis confirmed that all HMO-2011-type MVGs exhibit genomic synteny with HMO-2011-type phages (Fig. 1). Although some of these HMO-2011-type MVGs are highly similar to their cultivated relatives, most MVGs appear to have more genomic variations. To resolve the evolutionary relationship among the HMO-2011-type phages, a phylogenetic tree was constructed based on the concatenated sequences of five core genes. We found that HMO-2011-type phages are evolutionarily diverse and can be separated into at least 10 well-supported subgroups (>2 members), with 140 MVGs clustering into previously identified HMO-2011-type groups (subgroups I and III in Fig. 2A) [22], and the remaining 67 MVGs forming new subgroups (Fig. 2A). Among these HMO-2011-type subgroups, three contain cultivated representatives (subgroups I, III, and IX). Subgroup I contains the greatest number of phages, including six cultivated representatives and 123 MVGs (Fig. 2A). The cultivated representatives in subgroup I include a phage that infect SAR116 strain and five phages that infect *Roseobacter* strains. Subgroup III contains four cultivated representatives that infect two *Roseobacter* strains, and 17 MVGs. Pelagiphage HTVC033P and nine MVGs form subgroup IX. Other subgroups have no cultivated representatives yet. The results of phylogenomic analysis showed that subgroups I to VI are closely related, whereas subgroups VII to X are located on a separate branch and are more distinct from the subgroups I to VI, which suggests that these subgroups are more evolutionarily distant. A phylogenomic-based approach with GL-UVAB workflow [53] was also performed to cluster these HMO-2011-type genomes, which showed similar grouping results (Supplementary Fig. 2).

A previous study suggested the use of the percentage of shared proteins as a means of defining phage taxonomic ranks and proposed that phages with ≥20 and ≥40% orthologous proteins in common can be grouped at the taxonomic ranks of subfamily and genus, respectively [58]. Overall, most of the calculated percentages between HMO-2011-type genomes fall within the 20 to 100% range and most of the percentages between genomes within the same subgroup fall within the 40 to 100% range (Fig. 2B). Therefore, our results suggest that the HMO-2011-type is roughly a subfamily-level phage taxonomic group containing at least ten genus-level subgroups in the *Podoviridae* family.

### Conserved genomic structure and variation in HMO-2011-type phages

Of the 1235 orthologous protein groups (≥2 members) identified in HMO-2011-type genomes, only 254 proteins groups could be assigned putative biological functions (Supplementary Table 2). Comparative genomic analysis clearly revealed the conserved functional module structure of all HMO-2011-type genomes. All HMO-2011-type phage genomes can be roughly divided into the DNA metabolism and replication module, structural module and DNA packaging module (Fig. 1). Most of the homologous genes are scattered in similar loci of the HMO-2011-type genomes. Core genome analysis based on complete HMO-2011-type genomes revealed that HMO-2011-type genomes share a common set of ten core genes (Fig. 1). These core genes are mostly genes related to essential function in phage replication and development, including genes encoding DNA helicase, DNA primase, DNA polymerase (DNAP), portal protein, capsid protein, and terminase small and large subunits (TerL and TerS) as well as several genes with no known function, suggesting that phages in this group employ similar overall infection and propagation processes (Fig. 1).

Most members in subgroups I and III and one member in subgroup II possess a tyrosine integrase gene (*int*) located upstream of the DNA replication and metabolism module, whereas all subgroup IV to X genomes contain no identifiable lysogeny-related genes. This result suggests that members of subgroups IV to X might be obligate lytic phages. Integrase genes typically occur in the genomes of temperate phages and are responsible for site-specific recombination between phage and host bacterial genomes [59, 60]. In subgroup III, RCA phage CRP-3 has been experimentally demonstrated to be capable of integrating into the host genome [22]. Thus, certain *int*-containing HMO-2011-type phages are also likely to be temperate phages.

In the DNA metabolism and replication modules, genes encoding DNA primase, DNA helicase, DNAP, ribonucleotide reductase (RNR), and endonuclease can be identified; and DNA helicase, DNA primase, and DNAP are core to all HMO-2011-type phages. All reported HMO-2011-type phages contain an atypical DNAP, in which a partial DnaJ central domain is located between the exonuclease domain and the DNA polymerase domain [20, 22]. The *Escherichia coli* DnaJ protein, a co-chaperone [61], has been shown to be involved in diverse functions [62] and to be critical for the replication of phage Lambda [63–65]. The sequence analysis revealed that DNAP sequences of these seven new HMO-2011-type phages and 207 MVGs also present this unusual domain structure and contain two repeats of the CXXCXGXG motifs involved in zinc binding [66] in the partial DnaJ domain (Supplementary Fig. 3). RNR gene is frequently detected in subgroups I, II, III, IV, V, and X genomes but not in the other subgroup genomes. RNRs, which are widely distributed in diverse phage genomes, are involved in catalyzing the reduction of ribonucleotides to deoxyribonucleotides, and thus play a crucial role in providing deoxyribonucleoside triphosphates for phage DNA biosynthesis and repair [67–69]. RNR genes clustered with the RNR gene in phage HMO-2011 were previously reported to dominate the class II viral RNRs in examined marine viromes [69]. In the remaining two modules, genes involved in phage structure (e.g., genes encoding capsid and portal proteins), packaging of DNA (TerL and TerS genes), and cell lysis were detected. The proteins encoded by these genes play key roles in phage morphogenesis and virion release.

**Fig. 3:**
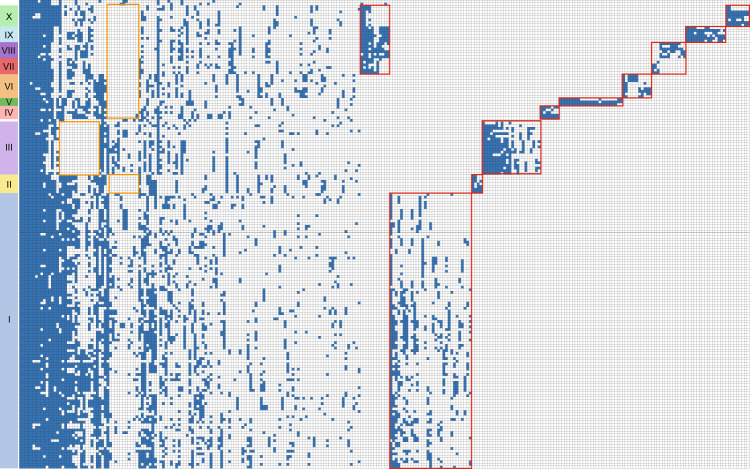
Distribution and functional classification of orthologous protein groups across HMO-2011-type genomes. Only orthogroups containing >10 members or showing subgroup-specific features are shown. Subgroup-specific genes are boxed in red. Genes that are absent in a specific subgroup are boxed in orange.

Examination of the distribution of the orthologous groups among the subgroups revealed clear pan-genome differences in various subgroups (Fig. 3). Most subgroups harbor subgroup-specific genes not identified in other subgroups, although  no function has yet been assigned to most of these genes. Notably, the phages in subgroups VII, VIII, and IX possess genomic features that differentiate them from phages in other subgroups, specifically with regard to the G + C content and gene content. The members of these three subgroups are closely related to each other in the phylogenetic tree and harbor several subgroup-specific genes. The G + C content of the phage genomes in these subgroups ranges from 31.9 to 35.4%, significantly smaller than other subgroups but similar to the G + C content of SAR11 bacteria and other known pelagiphages. HTVC033P is the only cultivated representative of subgroup IX. The aforementioned results suggest that the phages in subgroup VII, VIII, and IX might have related bacterial hosts and are highly likely to be pelagiphages. The host prediction using RaFAH tool also assigned *Pelagibacter* as their potential hosts (Supplementary Table 1). Subgroup X is located near these three subgroups in the phylogenetic tree, and the G + C content of the phages in this subgroup ranges from 34.4 to 39.0%. The host prediction assigned *Roseobacter* as their potential hosts. The hosts of this subgroup still remain to be experimentally investigated.

### Metabolic capabilities of HMO-2011-type phages

All HMO-2011-type phage genomes harbor several host-derived auxiliary metabolic genes (AMGs) potentially involved in diverse metabolic processes. Some AMGs in HMO-2011-type phages have been discussed previously [20, 22].

Subgroups VII, VIII, IX, and X possess distinct AMGs as compared with the other subgroups. For example, the genes encoding FAD-dependent thymidylate synthase (ThyX, PF02511) and MazG pyrophosphohydrolase domains are absent in all subgroups VII, VIII, IX, and X genomes but frequently detected in other subgroup genomes. ThyX protein is essential for the conversion of dUMP to dTMP mediated by an FAD coenzyme and is therefore a key enzyme involved in DNA synthesis [70, 71]. The *thyX* gene is commonly found in microbial genomes and phage genomes. Phage-encoded ThyX has been suggested to compensate for the loss of host-encoded ThyA and thus play crucial roles in phage nucleic acid synthesis and metabolism during infection [72]. Except in the case of subgroups VII, VIII, IX, and X genomes, the *mazG* gene, which encodes a nucleoside triphosphate pyrophosphohydrolase is sporadically distributed in HMO-2011-type genomes. MazG protein is predicted to be a regulator of nutrient stress and programmed cell death [73] and has been hypothesized to promote phage survival by keeping the host alive during phage propagation [74]. The *Escherichia coli* MazG can interfere with the function of the MazEF toxin–antitoxin system by decreasing the cellular level of (p)ppGpp [73]. However, a recent study showed that a cyanophage MazG has no binding or hydrolysis activity against alarmone (p)ppGpp but has high hydrolytic activity toward dGTP and dCTP, and it was speculated to play a role in hydrolyzing high G + C host genome for phage replication [75]. Whether the MazG proteins encoded by HMO-2011-type phages play a similar role in phage propagation remained to be investigated.

Five MVGs in subgroup I contain a gene encoding a DraG-like family ADP-ribosyl hydrolase (ARH). In cellular ADP-ribosylation systems, ARH catalyzes the cleavage of the ADP-ribose moiety, and thereby counteract the effects of ADP-ribosyl transferases [76]. It has been reported that ARH in *Rhodospirillum rubrum* regulates the nitrogen fixation [77]. However, the function of this phage-encoded ARH in the phage propagation process remains unclear.

We also observed that several MVGs possess genes involved in iron–sulfur (Fe–S) cluster biosynthesis, including an Fe–S cluster assembly scaffold gene (*iscU*) that involved in Fe–S cluster assembly and transfer [78] and an Fe–S cluster insertion protein gene (*erpA*). Fe–S cluster participates in a wide variety of cellular biological processes [79]. The discovery of these genes suggests that these phages may play important roles in Fe–S cluster biogenesis and function.

The gene encoding sodium-dependent phosphate transport protein (PF02690) has been identified in eight subgroup I genomes. The Na/Pi cotransporter family protein is responsible for high-affinity, sodium-dependent Pi uptake, and thus the protein plays a critical role in maintaining phosphate homeostasis [80]. This gene might function in the transport of phosphate into cells during phage infection. The presence of Na/Pi cotransporter genes suggests that some HMO-2011-type phages may have the potential to regulate host phosphate uptake in phosphate-limited ocean environments in order to benefit phage replication and propagation.

### Identification and phylogenetic analysis of HMO-2011-type DNAPs

The genetic diversity and geographically distribution of HMO-2011-type phages in marine environments was further inferred from DNAP gene analyses. A total of 2433 HMO-2011-type DNAP sequences with sequence sizes ranging from 540 to 779 amino acids were identified and subjected to phylogenetic analysis (Supplementary Table 3).

Among the identified HMO-2011-type DNAPs, 2030 sequences were retrieved from the GOV 2.0 *Tara* expedition upper-ocean viral populations (0–1000 m), from tropical to polar regions. HMO-2011-type DNAP genes were identified from all analyzed upper-ocean viromes, suggesting the global prevalence of HMO-2011-type phages in upper oceans.

**Fig. 4:**
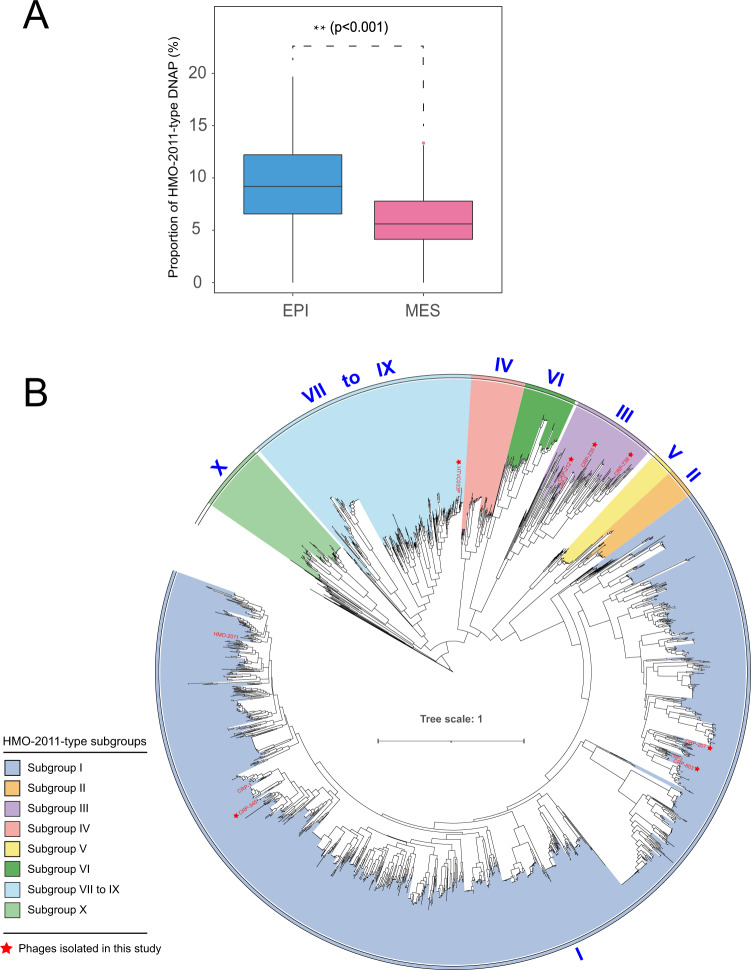
Proportion and phylogeny of HMO-2011-type DNAP sequences. **A** Box plot showing the estimated proportion of HMO-2011-type DNAPs to family A-type DNAPs in epipelagic and mesopelagic viromes. EPI epipelagic (<200 m), MES mesopelagic (200–1000 m). The pairwise comparison shown was statistically significant (*p* < 0.01) using two-tailed Mann–Whitney *U* tests. **B** Phylogenetic tree of HMO-2011-type DNAP sequences, shaded by subgroups. HMO-2011-type phage isolates are shown in red. Scale bar represents amino acid substitutions per site.

A previous study revealed that marine viromes contain various types of tailed phage genomes that encode a family A DNAP gene [81]. To estimate the importance of HMO-2011-type phages, we calculated the proportion of HMO-2011-type DNAPs based on the number of HMO-2011-type DNAP sequences and the total number of family A DNAP sequences (>470 aa) in each GOV 2.0 viral population dataset. This analysis revealed that HMO-2011-type DNAPs accounted for up to 19.7% of all family A DNAPs in each GOV 2.0 dataset (Supplementary Table 4). We found that the HMO-2011-type DNAP sequences appear to be more dominant in epipelagic viromes than in mesopelagic viromes (*p* < 0.001, Mann–Whitney *U* tests) (Fig. 4A), and that the proportion of HMO-2011-type DNAPs positively correlated with temperature *(p* < 0.01; *R*^2^ = 0.11). These results further demonstrate that the HMO-2011-type group is numerically abundant and widespread across the world’s oceans.

The phylogenetic tree established using all the identified HMO-2011-type DNAPs shows a largely consistent topology with the phylogenetic tree constructed using concatenated five core genes of all HMO-2011-type phages, except that subgroups VII, VIII, and IX do not show clear separation (Fig. 4B). A recent study identified two MVGs that contain the HMO-2011-type DNAP and Cobavirus-type structural and packing genes [82]. We found the DNAPs closely related the DNAPs of these two MVGs are located on different branches that are distinct from these identified subgroups. In the DNAP tree, 67.6% of the DNAP sequences are classified into subgroup I with geographically diverse origins, indicating that subgroup I is the largest subgroup and is geographically widespread in the ocean. Most of the DNAP sequences in this subgroup were originated from epipelagic zones in distinct ocean regions, from tropical to polar stations. Certain subgroups show distribution pattern related to temperature. For example, subgroup II, IV, and V were dominated by DNAP sequences from tropical to subtropical stations, where temperatures were normally >20 °C. By contrast, subgroup III mostly comprised of DNAP sequences from temperate to polar stations, where temperature were normally <20 °C. Subgroups VII–IX contain 12.8% of all the identified DNAP sequences, and the DNAPs in these subgroups were also widespread. Taken together, this DNAP survey further revealed that highly diverse and abundant HMO-2011-type DNAP sequences were prevalent in marine environments.

### Global distribution of HMO-2011-type phages

**Fig. 5:**
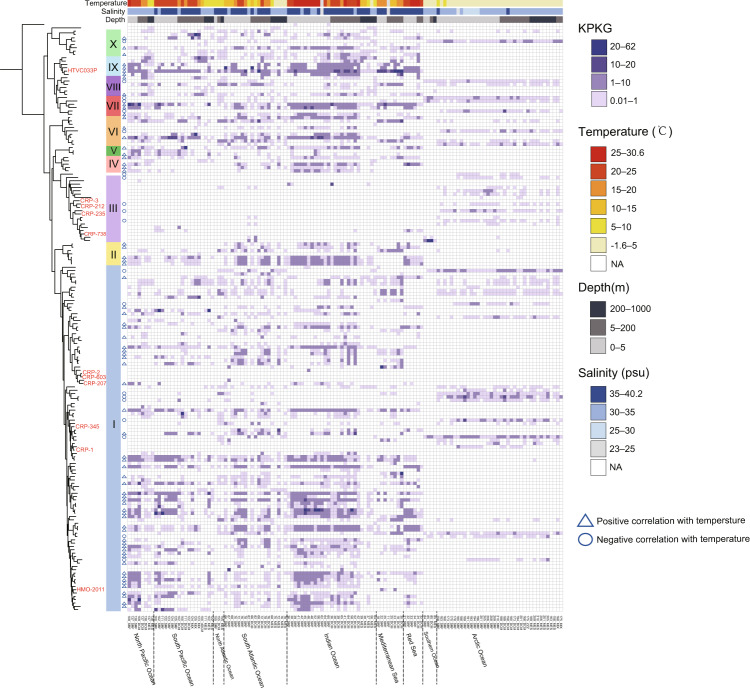
Heatmap displaying relative abundance of each HMO-2011-type phages in different marine viromic datasets. Normalized relative abundance is depicted as total recruited nucleotides (kb) per kilobase of genome per gigabase of metagenome (KPKG).

The HMO-2011-type phage group has been demonstrated to be among the most abundant known phage groups in most marine viromes [20, 22]; however, the relative abundance of each HMO-2011-type genome and the distribution patterns of distinct HMO-2011-type subgroups remain poorly elucidated. Therefore, we performed metagenomic read recruitment at the species-level (≥95% nucleotide identity) by mapping reads to each HMO-2011-type genome (Fig. 5). Viromic reads mapped to these HMO-2011-type genomes were present in all epipelagic and mesopelagic viromes (0–1000 m) with varying relative abundance, and attributed up to 0.9% of the total reads (Supplementary Table 5). By contrast, neither genome was detected in deep ocean viromes (>1000 m). This observation was as expected because all HMO-2011-type phages were isolated from the upper ocean, and all HMO-2011-type MVGs were identified from upper-ocean viromes.

Among all identified HMO-2011-type phages, many phages were prevalent and more abundant in the higher temperature tropical and temperate regions. Linear-regression analysis showed that there was a positive correlation with temperature (*p* < 0.01; *R*^2^ = 0.03–0.46) (Supplementary Table 6). However, this pattern is strongly contrasted in the case of certain HMO-2011-type phages originated from polar viromes (Fig. 5). These phages occupied Arctic and Antarctic systems and showed a negative correlation with temperature (*p* < 0.01; *R*^2^ = 0.07–0.31) (Supplementary Table 6). Moreover, some of the HMO-2011-type phages were detected prevalent in both cold and warm stations and showed no significant correlation with temperature, which suggests that they may infect host that have broader distribution or can infect both cold- and warm-type hosts. We also noticed that the abundance of some MVGs display significant correlations with various parameters (Supplementary Table 6).

We observed that phages within the same subgroup can present distinct distribution pattern. At the subgroup level, subgroup I contains most members. Subgroup I members were mostly detected in the epipelagic zone of tropical and temperate regions (0–200 m) and were also detected in polar stations (Fig. 5). The reads assigned to the current identified subgroup I members account for 56.8% of the total reads assigned to the entire HMO-2011-type group. However, it should be noticed that this analysis only includes identified HMO-2011-type phages; additional HMO-2011-type phages that are more abundant potentially remain to be discovered. Although most members in subgroup I were widely distributed and have relatively higher KPKG values, all cultivated representatives in this subgroup were found to be either absent or only detected in limited stations and have very low KPKG values (Fig. 5), suggesting that the most abundant members in this subgroup have not yet been isolated. Subgroup III, represented by RCA phage CRP-3 and two other roseophages, is one of the least abundant subgroups. Subgroup III members were present mostly in polar stations, where the temperatures were low, and this agrees with the distribution pattern of subgroup III DNAPs. Subgroup II, IV, V, and IX were frequently detected in tropical and temperate regions but were absent in all polar stations, suggesting that the hosts infected by these phages displayed a limited distribution and might not be able to adapted to the cold-water environments. Subgroup IX members were frequently detected with relatively higher KPKG values and displayed similar patterns. HTVC033P was overall the most abundant known HMO-2011-type phage, followed by several MVGs in subgroup IX (Fig. 5). The highest KPKG values of HTVC033P occurred at the stations located in the Mediterranean Sea, from which it was originally isolated. Subgroups VII and VIII phages, which are closely related to subgroup IX were detected in both warm and cold regions. Some phages in subgroup VII and VIII were prevalent in polar stations, suggesting that their hosts can adapt to cold-water environments.

**Fig. 6:**
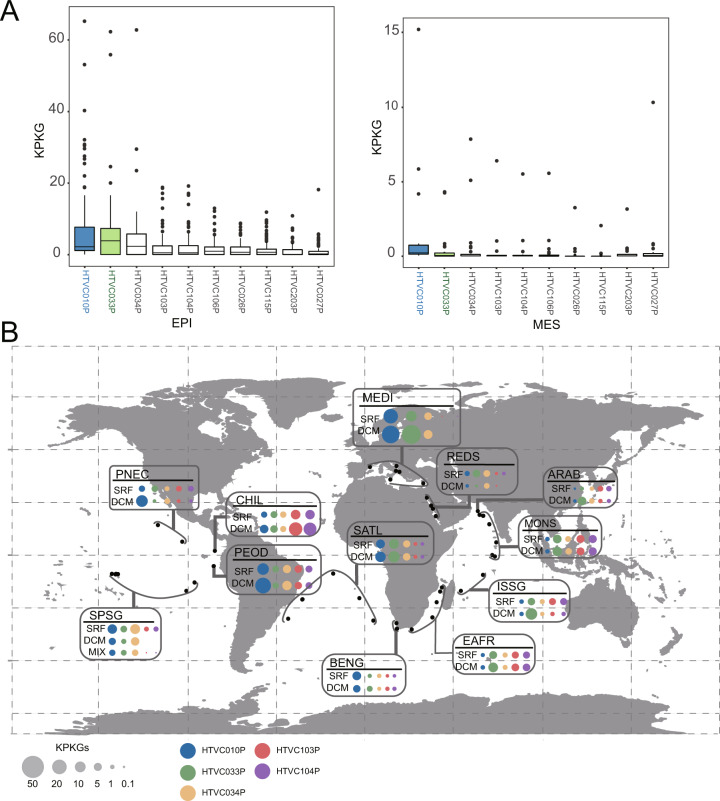
Comparison of HTVC033P with other pelagiphages. **A** Box plots indicating relative abundances of the ten most abundant pelagiphages occurring in at least 50% of the viromes. Normalized relative abundance is depicted as KPKG. The newly isolated pelagiphage HTVC033P and the previously reported pelagiphage HTVC010P are shown in green and blue, respectively. EPI epipelagic (<200 m), MES mesopelagic (200–1000 m). **B** Biogeography of the five most abundant marine pelagiphages. Bubbles represent the relative abundances expressed in KPKG. MEDI Mediterranean Sea, REDS Red Sea, ARAB NW Arabian Upwelling, MONS Indian Monsoon Gyres, ISSG Indian S. Subtropical Gyre, EAFR E. Africa Coastal, BENG Benguela Current Coastal, SATL S. Atlantic Gyre, PNEC N. Pacific Equatorial Countercurrent, SPSG S. Pacific Subtropical Gyre Province, CHIL Chile-Peru Current Coastal Province.

In comparison with other previously reported pelagiphage isolates, we found that HTVC033P is among the most abundant pelagiphage isolates. HTVC033P was found to be generally less abundant than HTVC010P, but more abundant than other pelagiphages in both epipelagic and mesopelagic viromes (Fig. 6A). In terms of distinct oceanic regions, our findings indicate that HTVC033P is the most abundant pelagiphage in the Red sea, Indian Ocean and South Atlantic, and the second or third most abundant pelagiphage in the Pacific Ocean (Fig. 6B). These results suggests that HMO-2011-type pelagiphages are a biologically and ecologically important type of pelagiphages.

## Conclusions

The discovery of HMO-2011-type phages in the world’s oceans raised several questions regarding to their diversity, ecology, and roles in microbial communities. Here, we performed a culture-based and metagenomics-based analysis of the genomic diversity and distribution of the HMO-2011-type phage group. The obtained HMO-2011-type genomes help reveal the genuine extent of the genetic diversity of HMO-2011-type phages within natural populations of marine viruses. Our findings show that the HMO-2011-type group contains diverse subgroups that might infect distinct bacterial groups, which highlights the critical roles of HMO-2011-type phages in the world’s oceans. Furthermore, metagenomic mapping of the HMO-2011-type phages has revealed several distinct distribution patterns related to water temperature. These novel insights into the diversity and ecology of HMO-2011-type phages further expanded current understanding of these important phages. Lastly, further investigation using our newly constructed virus–host models will provide additional valuable insights into the influence of viruses on the function and diversity of ocean microbial communities, and carbon biogeochemistry.

## Supplementary information

Supplementary Fig. 1

Supplementary Fig. 2

Supplementary Fig. 3

Supplementary Table 1

Supplementary Table 2

Supplementary Table 3

Supplementary Table 4

Supplementary Table 5

Supplementary Table 6

## Data Availability

The seven new phage isolates were deposited in GenBank under the accession numbers MZ892987 to MZ892993.
