# Supplementary material for: Newly identified HMO-2011-type phages reveal genomic diversity and biogeographic distributions of this marine viral group
Source: ISME J. 2022 Jan 12;16(5):1363–75. doi: 10.1038/s41396-021-01183-7 (PMC9038755; doi:10.1038/s41396-021-01183-7)
Supplement: Supplementary file 1 — Supplementary Fig. 1 [file 41396_2021_1183_MOESM1_ESM.pdf]

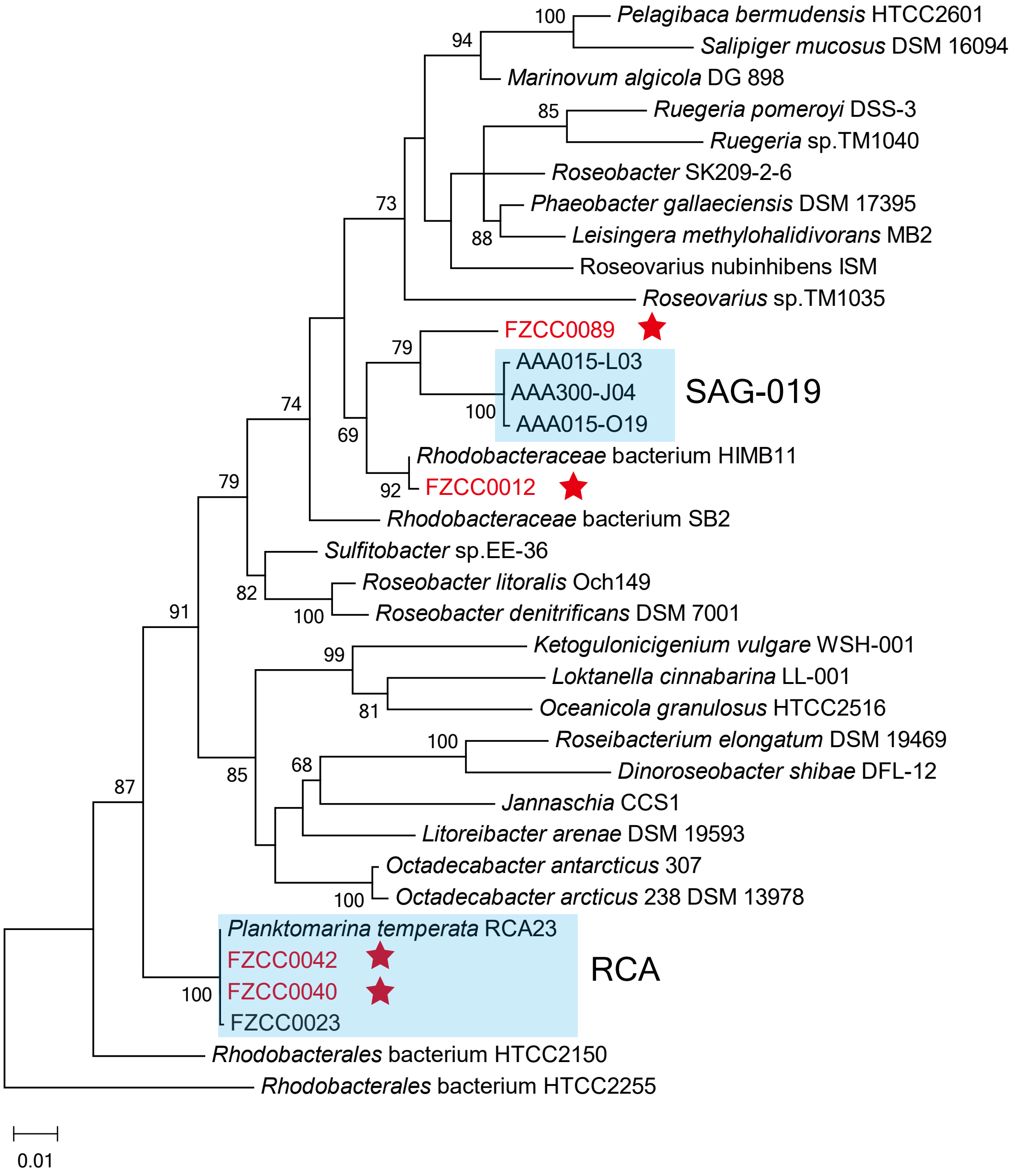

Supplementary Fig. 1 The phylogenetic position of the *roseobacter* hosts based on 16S rRNA gene sequence analysis. *Roseobacter* strains used for phage isolation were indicated with red asterisks.
