# Supplementary material for: Newly identified HMO-2011-type phages reveal genomic diversity and biogeographic distributions of this marine viral group
Source: ISME J. 2022 Jan 12;16(5):1363–75. doi: 10.1038/s41396-021-01183-7 (PMC9038755; doi:10.1038/s41396-021-01183-7)
Supplement: Supplementary file 2 — Supplementary Fig. 2 [file 41396_2021_1183_MOESM2_ESM.pdf]

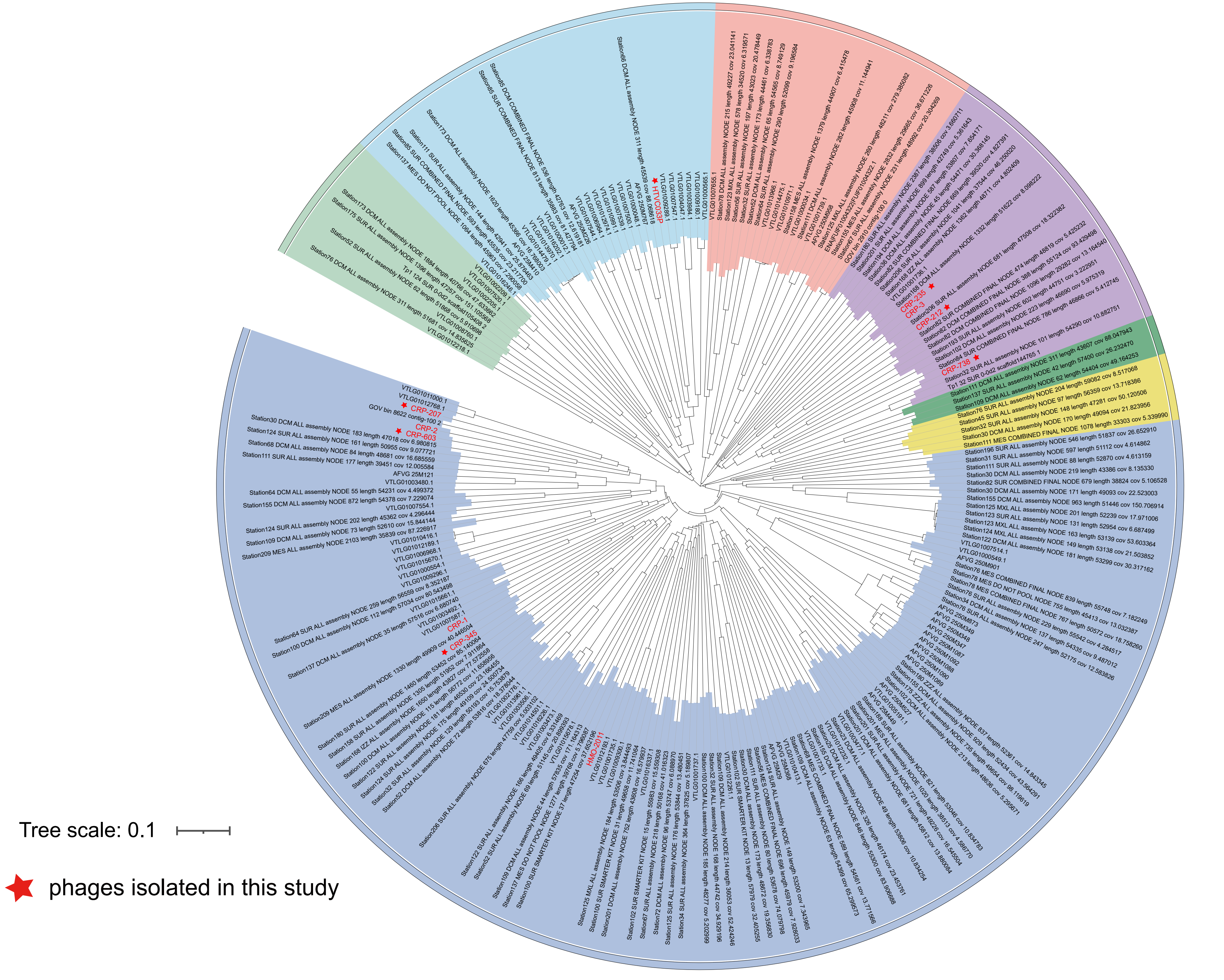

Supplementary Fig. 2 Phylogenomic tree constructed based on Dice distances matrix with the GL-UVAB workflow. The taxonomic classification of HMO-2011-type phages at the genus level was performed according to the recommended minimum node depth of 0.0189 and number of representatives equal or above 3.
