# Supplementary material for: Newly identified HMO-2011-type phages reveal genomic diversity and biogeographic distributions of this marine viral group
Source: ISME J. 2022 Jan 12;16(5):1363–75. doi: 10.1038/s41396-021-01183-7 (PMC9038755; doi:10.1038/s41396-021-01183-7)
Supplement: Supplementary file 3 — Supplementary Fig. 3 [file 41396_2021_1183_MOESM3_ESM.pdf]

CXXCXGX motif CXXCXGX motif

VTLG01016246_9	RTKVHKCKVCNGWGKYYKKKKDGTNYKKQHNCKACEGKGAGL
Station158_MES_ALL_assembly_NODE_1379_length_44907_cov_6.415478_10	KVRAVACTDCKGFGKAYKIKKDGSNFAKPSKCLTCEGRGAGL
Station52_DCM_ALL_assembly_NODE_65_length_54565_cov_8.749129_36	KTRSLQCPGCHGRGTFFKTKKNGERFKKATKCQMCDGRGAGL
VTLG01001739_33	KTQAVRCLYCRGTGSIQKYKKNGTPYKNISKCIECDASGAGL
AFVG_250M958_57	KTQAVRCLYCRGTGSIQKYKKNGTPYKNISKCIECDASGAGL
VTLG01000034_33	KTQAVKCPHCFGKGGYWRIKKDGTRFKNMTKCDHCDGTGAGL
Station111_DCM_ALL_assembly_NODE_282_length_45908_cov_1144941_36	KTQAKRCLFCNGKGTIQKYKKDGTPYKNTTKCVHCEGTGAGL
GOV_bin_2910_contig-100_0_23	KTRVIKCGSCYGKGTYYKLKKDGSNFKKPSKCEYCEGLGAGL
Station67_SUR_ALL_assembly_NODE_231_length_48992_cov_20.304269_41	KTRVIKCGSCYGKGTYYKLKKDGSNFKKPSKCEYCEGLGAGL
Station155_MES_ALL_assembly_NODE_2832_length_29665_cov_36.671226_10	KTRVIKCGSCHGKGTYYKVKKDGNNFKKPSKCWYCQGLGAGL
FUIF01004322_1	KVRAIKCTACDGRGEYYKLKKDGSNFKKATKCPHCLGKGGGL
Station125_MXL_ALL_assembly_NODE_260_length_48211_cov_279.385082_1	KVRAIKCAACDGRGEYYKVKKDGSNFKKATKCPHCLGKGGGL
Station180_SUR_ALL_assembly_NODE_2387_length_38506_cov_3.660711_32	RTKGVQCPTCLGVGTTQKIKKNGQDYKNRSKCVPCSSIGAGF
VTLG01001736_49	KQVASHCRACNSIGTIRKIKKDGTPYKNVSKCESCKGLGAGL
Station194_DCM_ALL_assembly_NODE_587_length_53807_cov_7.654171_47	KTNAEHCNTCEGKGYFYKFKANGERWKKSTKCVACEGRGAGL
Station201_SUR_ALL_assembly_NODE_899_length_42749_cov_5.361643_34	KTKAEHCITCKGRGQYLKTKKNGEPYKKASTCAVCDGRGAGF
Station193_SUR_ALL_assembly_NODE_602_length_44751_cov_3.222951_17	KTMAIRCMDCNGIGTIQNYKKQGEPYKNRNKCKTCKGLGAGL
Station82_DCM_COMBINED_FINAL_NODE_1096_length_29282_cov_1364540_6	KTMASRCVDCNGIGSIQQYKMQGEAYKNRTKCKTCKGLGAGL
Station102_DCM_ALL_assembly_NODE_223_length_46690_cov_5.975319_54	KTQAVTCPDCNGRGDFQQYKTKGDPYKNRTKCKPCDGKGGGL
Station82_DCM_COMBINED_FINAL_NODE_388_length_55124_cov_93.429498_40	RTQASQCPDCRGAGSIQKYKVTGEAYKNRTKCAVCDGAGAGL
Station82_SUR_COMBINED_FINAL_NODE_474_length_48819_cov_5.425232_42	KTQAVKCSDCNGIGSIQKFKVQGDPYKNRTKCKTCSGVGGGL
CRP-738	RKTAHKCRDCNGVGSIQKFKQQGEPYKNRTKCKVCDGVGAGL
Station32_SUR_ALL_assembly_NODE_101_length_54290_cov_10.882751_36	KTVASRCQCCDGLGSIQKFKQQGGPYKNRTKCKTCNGKGAGL
Tp1_32_SUR_0-0d2_scaffold144765_1_13	KTVASRCQCCDGLGSIQKFKQQGGPYKNRTKCKTCNGKGAGL
CRP-235	RTMARCCDTCDGRGKIQKVKKNGELWKNLSKCANCNGVGAGL
CRP-3	RTVAHHCTECDGKGKIQKIKKDGKPYKQLTKCPVCQGEGAGL
CRP-212	RTVAMCCKECEGYGTIQKFKKNGDPWKNRSKCPVCKGAGAGL
Station206_SUR_ALL_assembly_NODE_681_length_47508_cov_18.322382_33	RTVAMCCQLCNGIGTVQKIKKDGEPWKVRSKCSICEGLGAGL
Station82_SUR_COMBINED_FINAL_NODE_669_length_39520_cov_4.827391_37	KTIASQCPNCKGRGYYQKYKKNGDPWAKTSNCKDCDTLGAGL
Station168_IZZ_ALL_assembly_NODE_1362_length_48711_cov_4.802409_57	KTVGYHCDLCKGKGKIQKYKKDGTPWINLTKCQPCDGQGAGL
Station36_DCM_ALL_assembly_NODE_45_length_54471_cov_30.368145_42	QTVAEHCHRCKGTGKIQKFKKDGTPWKNKSKCPACNGAGAGL
Station206_SUR_ALL_assembly_NODE_1041_length_37544_cov_46.250020_17	RTIANHCDVCGGEGRLQKTKVNGDLYKNLSKCTHCDGRGAGL
Station111_DCM_ALL_assembly_NODE_311_length_43607_cov_88.047943_59	QTTVRQCPNCKGTGKQHKIKKDGTPYKVQPMCKHCLGTGAGF
Station109_DCM_ALL_assembly_NODE_62_length_54404_cov_4964253_14	KTTVRKCPDCKGTGKQYKVKKDGSPYKIQPMCKHCIGTGAGF
Station137_SUR_ALL_assembly_NODE_42_length_57400_cov_26.232470_66	KTVVRKCPDCKGTGKQYKVKKDGSPYKIQPMCKHCIGTGAGF
Station137_MES_DO_NOT_POOL_NODE_1064_length_45963_cov_7.290058_13	KTEAIHCKDCDGLGYLYKITQSGVPYKNQPKCKVCSGVGAGF
VTLG01012218_52	RTKGTPCSNCNGYGKIRGYTKSGALSKVQKTCKECDGIGAGL
Station76_DCM_ALL_assembly_NODE_311_length_51681_cov_14.835625_42	KTRGKPCDHCGGKGKIRPYTKSGVLSKVQKTCKKCGGKGAGL
Station173_DCM_ALL_assembly_NODE_1884_length_40766_cov_47.633662_39	HTESHQCGSCRGRGYFNPLKKDGTAGKAKRICKTCNSEGGGF
Station175_SUR_ALL_assembly_NODE_1396_length_47257_cov_15105568_3	HTESHQCGSCRGRGYFNPLKKDGTAGKAKRICKTCNSEGGGF
Tp1_124_SUR_0-0d2_scaffold105408_2_57	QTESQRCGDCQGKGYIHPLKKDGTIGKAKRICKTCDGQGGGF
VTLG01002209_52	KTRMQRCTACGGIGYQYALKRDGTVGKQKRICKVCNKKGAGL
VTLG01007520_14	KTRMQRCTACGGIGYQYALKRDGTVGKQKRICKVCNKKGAGL
VTLG01002205_16	KTVMRRCTACGGIGYKYALKRDGTVGKQKRICVVCSKKGAGL
VTLG01008760_1	KTRVNRCDSCNGLGHFFALKKDGTVGKQRRICKSCTGKGAGF
Station52_SUR_ALL_assembly_NODE_62_length_51868_cov_5.910698_19	KTKMESCNKCGGSGYFYAMKKDGTIGKQKRICKACRGKGAGF
Station76_SUR_ALL_assembly_NODE_204_length_59082_cov_8.517068_17	KTRVEQCGKCSGKGDFYRVKKDGSLWKKSTRCKACGGEGGGL
Station45_SUR_ALL_assembly_NODE_97_length_56359_cov_13.718386_13	KTRAEQCGNCKGKGEFYKIKKDGSRWKNATRCKSCGGDGGGL
Station32_SUR_ALL_assembly_NODE_148_length_47281_cov_5020506_40	KTRASHCKSCKGIGTYTKVKKDGKPYKKPTKCSVCVGKGGGL
Station30_DCM_ALL_assembly_NODE_170_length_49094_cov_21.823956_22	KTSAQHCYKCNGKGEFYKTKKDGTRWKKPSKCPPCQGQGAGL
Station111_MES_COMBINED_FINAL_NODE_1078_length_33303_cov_5.339990_8	KTKARQCTQCFGKGSIHKIKKDGTRWKNPTKCKPCSGRGGGL
Station85_SUR_COMBINED_FINAL_NODE_593_length_45535_cov_23.217700_52	YTIGTKCNVCKGVGAYRKTKVDGKPFLNITKCADCSGHGAGF
AFVG_25M410_58	KTIANDCPECQGLGLVRHTKVDGTPFKSMNKCKKCKSLGAGF
Station111_SUR_ALL_assembly_NODE_144_length_42941_cov_25.876463_59	KTMATQCKTCNGVGLVRHTKVDGTPFKNMSKCSPCKGEGAGF
VTLG01014479_33	KTKSEQCKECQGKGKVQKLKVDGTPYKNLTKCDKCDGGGAGF
VTLG01013970_44	KTKSEQCKECQGKGKVQKLKVDGTPYKNLTKCDKCDGGGAGF
VTLG01000012_23	KTKSEQCKECQGKGKVQKLKVDGTPYKNLTKCDKCDGGGAGF
VTLG01016202_23	KTKSEQCKECQGKGKVQKLKVDGTPYKNLTKCDKCDGGGAGF
Station173_DCM_ALL_assembly_NODE_1620_length_45366_cov_16.768003_21	KTKSEQCPECHGKGKIQKVKVDGSNYVNLSKCPTCVGDGAGF
AFVG_250M226_22	KTKAIRCGECLGKGYIQKIKVNGDPYKNLSKCSRCKSEGAGF
Station111_DCM_ALL_assembly_NODE_317_length_43419_cov_13.731621_40	KTKAKQCTECSGKGYIQKYKVNGDKYKNMSKCPVCKSEGAGF
Station85_DCM_COMBINED_FINAL_NODE_536_length_42763_cov_12.619181_17	KTKAEHCVACSGKGQIQKFKVNKEPYKKLTKCFDCGGKGAGF
VTLG01006964_15	KTKAHQCTTCKGRGVVQKIKVNGEPYKNLSKCVDCNAQGAGF
VTLG01007544_9	KTKAHQCTTCKGRGVVQKIKVNGEPYKNLSKCVDCNAQGAGF
Station85_SUR_COMBINED_FINAL_NODE_813_length_35883_cov_81.427794_15	KTVADQCTACSGDGTMQRRKVNGDVYKNLSRCDVCQGQGAGF
VTLG01007547_41	KTNATQCIGCKGKGVVKKIKKDGTPFKKYSKCSECSGEGAGF
VTLG01009289_64	KTNATQCIGCKGKGVVKKIKKDGTPFKKYSKCSECSGEGAGF
Station66_DCM_ALL_assembly_NODE_311_length_45539_cov_88.068618_50	KTSASKCLACDGKGVIKRIKKDGSPYKNYTKCSDCDGDGAGF
HTVC033P	RTVAEQCVHCKGKGVIKKIKKDGSPFKNYTKCPECDGEGAGF
VTLG01000565_15	KTVAEKCVTCGGKGVIKRIKKDGSPFKNYSKCSDCDGDGAGF
VTLG01009180_30	KTVAEKCVTCGGKGVIKRIKKDGSPFKNYSKCSDCDGDGAGF
VTLG01000048_24	KTTASQCESCKGKGVIHKIKVDGTPYKKYTKCAVCDGDGAGF
VTLG01003984_91	KTTANQCIDCKGKGVIKKMKVDGTPYKKYSKCIYCDGEGAGF
VTLG01004047_1	KTTANQCIDCKGKGVIKKMKVDGTPYKKYSKCIYCDGEGAGF
VTLG01007500_30	KTQSEQCPSCNGRGVYRKYKKDGEPYKNTTKCEQCNGEGAGF
VTLG01007539_68	KTQSEQCPSCNGRGVYRKYKKDGEPYKNTTKCEQCNGEGAGF
VTLG01012974_14	KTTASQCNSCHGKGHIQKIKVNGEPYKNLSKCETCVGQGAGF
VTLG01010991_64	KTKAIQCSDCSGKGVYQKYKVNGDPYKNLSKCETCTGQGAGF
VTLG01014475_40	RTFAVHCTACDGTGKVFKTRKDGTKYARGHTCAECGGVGAGF
VTLG01010971_4	RTFAVHCTACDGTGKVFKTRKDGTKYARGHTCAECGGVGAGF
VTLG01013966_55	RTFAVHCTACDGTGKVFKTRKDGTKYARGHTCAECGGVGAGF
Station78_DCM_ALL_assembly_NODE_215_length_49227_cov_23.041141_32	KTIASHCNHCNGTGRLRKLRKDGSPYKNEHTCSYCGGSGAGL
VTLG01007655_30	KTVATHCHTCDGSGKLRKTRKDGSPYVKAHTCYRCGGAGAGL
AFVG_250M1087_17	KTTAVSCADCQGKGYNLYVKKDGTVGKAKRICRTCNHKGAGL
AFVG_250M873_19	KTTAVSCADCQGKGYNLYVKKDGTVGKAKRICRTCNHKGAGL
AFVG_250M1090_54	KTTAVSCADCQGKGYNLYVKKDGTVGKAKRICRTCNHKGAGL
AFVG_250M1089_18	KTTAVSCADCQGKGYNLYVKKDGTVGKAKRICRTCNHKGAGL
AFVG_250M1092_20	KTTAVSCADCQGKGYNLYVKKDGTVGKAKRICRTCNHKGAGL
AFVG_250M1088_20	KTTAVSCADCQGKGYNLYVKKDGTVGKAKRICRTCNHKGAGL
AFVG_250M347_55	KTTAVSCADCQGKGYNLYVKKDGTVGKAKRICRTCNHKGAGL
AFVG_250M349_18	KTTAVSCADCQGKGYNLYVKKDGTVGKAKRICRTCNHKGAGL
AFVG_250M348_20	KTTAVSCADCQGKGYNLYVKKDGTVGKAKRICRTCNHKGAGL

CXXCXGX motif CXXCXGX motif

Station30_DCM_ALL_assembly_NODE_219_length_43386_cov_835330_16	KTHSEMCKTCKGNGHYYKVKKDGTKYKKSTTCPDCKGRGAGL
Station124_SUR_ALL_assembly_NODE_202_length_45362_cov_4.296444_3	KTKAIKCDECNGNKKIHKVKKDGTLFKKPNICKKCSGEGAGL
Station155_DCM_ALL_assembly_NODE_872_length_54378_cov_7.229074_58	KTKARQCDECRGEKKVFKIRKDGTLFKKANICKRCTGNGGGL
VTLG01007554_9	KTKARQCEECKGEKKIFKVKKDGTLFKKANICKTCMGNGGGL
Station64_SUR_ALL_assembly_NODE_259_length_56559_cov_8.352187_31	KTEAYHCKECNGKGYVHKRKKDGSLFKKPNRCKECDTRGAGL
Station209_MES_ALL_assembly_NODE_2103_length_35839_cov_87.226917_54	KTKAHRCDECKGNKYVHKTRKDGSLFKKPTKCKSCDATGAGL
VTLG01010416_44	KTKARTCDECDGKGKVHKKKKDGTPFAKPNKCKKCDAKGAGL
VTLG01006968_58	KTKARTCDECDGKGKVHKKKKDGTPFAKPNKCKKCDAKGAGL
VTLG01012189_39	KTKARTCDECDGKGKVHKKKKDGTPFAKPNKCKKCDAKGAGL
VTLG01009296_60	KTKASVCQKCNGKGTIRKIKKDGNPFAKPSRCPECDTRGAGL
VTLG01000554_34	KTKASVCQTCNGKGKVFKTKKDGQPFAKPSRCVDCNTRGAGL
Station30_DCM_ALL_assembly_NODE_183_length_47018_cov_6.980815_49	KTRCFTCPACEGEGKVYKIKKDGTRYAKPNKCKDCLGRGAGL
CRP-603	RTKAFTCPTCNGEGKTYKVKKDGTRFKKPNNCKDCDARGAGL
CRP-2	RTKAFTCPTCNGEGKTYKVKKDGTRFKKPNNCKDCDARGAGL
VTLG01011000_45	RTQAYTCPSCEGQGKVYRFKKDGTKFARPNKCKDCNALGAGL
VTLG01012768_52	RTQAYTCPSCEGQGKVYRFKKDGTKFARPNKCKDCNALGAGL
CRP-207	RTKAFTCPTCEGQGKTYKTKKDGTRFAKPNKCKDCDTRGAGL
GOV_bin_8622_contig-100_2_1	RTKAFTCPTCEGVGKVFKLKKDGTKYARPNRCKDCDTRGAGL
Station111_SUR_ALL_assembly_NODE_177_length_39451_cov_12.005584_15	KTYAVTCPTCKGKGKTYKVRKDGTKYAKPNKCKDCDARGAGL
Station124_SUR_ALL_assembly_NODE_161_length_50955_cov_9.077721_46	KTYAVICSSCTGKGKVFKVRKDGTKYVKPTKCKECDSKGAGL
Station68_DCM_ALL_assembly_NODE_84_length_48681_cov_16.685559_20	KTHAVICASCVGKGKVFKVRKDGTKYAKPTKCKECDGKGAGL
VTLG01003480_37	KTEANTCPECDGTGKVYKTKKDGTRYARPHKCPKCDGKGAGL
AFVG_25M121_18	KTEANTCPECDGTGKVYKIKKDGTRYARPHKCPKCDGKGAGL
Station64_DCM_ALL_assembly_NODE_55_length_54231_cov_4.499372_27	KTKASTCPTCNGGGRVYKTRKDGTLYKIPNKCKDCDGRGAGL
Station196_SUR_ALL_assembly_NODE_546_length_51837_cov_26.652910_18	KTKAIKCKECRGNGLFYKTKKNGSDFKNPSKCPTCVGRGAGL
Station111_SUR_ALL_assembly_NODE_88_length_52870_cov_4.613159_11	KTVAKQCSACKGKGTYYKKKKDGSNFKKPSKCATCNGAGAGL
Station31_SUR_ALL_assembly_NODE_597_length_51112_cov_4.614862_19	KTKAEQCKDCKGNGTYYKKKKNGDNFKNASKCSVCNGTGAGL
AFVG_250M901_22	KTVASKCSVCYGNGKVRKTRKDGKPFTKATKCGACDGEGAGL
Station34_SUR_ALL_assembly_NODE_364_length_37825_cov_589807_14	KTKAKQCPSCKGHGKYRKTKKDGTPFANETKCPTCLATGAGL
Station100_SUR_SMARTER_KIT_NODE_31_length_49658_cov_11.741064_57	KTLAEKCNICNGKGYVRKTKKDGTPYAKDNKCIACKADGAGL
VTLG01007587_45	KKKAFRCEACDGKGFIQRIRKDGKPYAKMSKCSVCDSQGAGL
VTLG01007514_58	KTKVRQCKDCSGAGRKRVRKKDGTLGKAVRICQTCGGEGAGL
VTLG01000549_24	KTKVRQCKDCSGAGRKRVRKKDGTLGKAVRICQTCGGEGAGL
Station201_SUR_ALL_assembly_NODE_721_length_49226_cov_16.545504_14	KSYVKRCEDCYGSGKIRKEKKNGTPYTKMSKCVSCSSHGGGL
Station201_DCM_ALL_assembly_NODE_681_length_45812_cov_13.880084_50	KSYVKRCEDCYGSGKIRKEKKNGTPYTKMSKCVSCSSHGGGL
Station201_MES_ALL_assembly_NODE_1020_length_38513_cov_4.586770_50	KSYVKRCEDCYGSGKIRKEKKNGTPYTKMSKCVSCSSHGGGL
VTLG01005191_49	KASLKQCKTCYGSGKIRKVKKDGNPFAKETRCPTCGGNGAGL
AFVG_25M449_27	KASLKQCKTCYGSGKIRKVKKDGNPFAKETRCPTCGGNGAGL
AFVG_250M527_31	KAKLQKCSVCYGSGKIRKERKNGLPYKKATKCSFCSGNGAGL
VTLG01001733_55	KTKAVRCNICNGKGEIRKTKKNGLPYAKNSKCHDCNSLGAGL
VTLG01003477_63	KTKAIRCQKCDGKGKIRKIRKDGTPFAKENKCPTCNSLGAGM
VTLG01012232_21	KTEAKKCVDCKGKGTIRKVKKDGNPYSRDSKCHTCNSLGAGL
VTLG01003492_46	KKRAIKCSSCYGSGYVRKKKKDGTPFANRNRCIACDAVGAGL
VTLG01015661_51	KQKAHQCTACKGTGSVRKVKKDGSLFARSNKCNDCTGIGAGL
Station100_DCM_ALL_assembly_NODE_112_length_57034_cov_80.543498_25	KQKAHQCTACKGTGSVRKVKKDGKLFARSNKCNDCTGIGAGL
Station52_DCM_ALL_assembly_NODE_72_length_53816_cov_18.378044_52	KTQARQCNDCNGTGKTYKTRKDGSRYAKPNHCKDCGGGGAGL
VTLG01003506_3	KQKAERCYECSGTGQIRKIRKNGTPHKNTNKCPVCSASGAGL
Station206_SUR_ALL_assembly_NODE_675_length_47759_cov_5.003102_45	KQKAVQCKGCEGTGQIRKTRKNGTPFTNTTKCSPCNGGGAGL
Station168_IZZ_ALL_assembly_NODE_1650_length_43827_cov_77.572558_17	KQKAYQCHDCRGSGRIHRTKKDGTPFAKASKCTTCVAHGAGL
Station158_SUR_ALL_assembly_NODE_1305_length_51952_cov_7.911864_60	KQKAHQCKECYGTGQVRKVKKDGTPFARTNKCNTCTGSGAGL
Station32_SUR_ALL_assembly_NODE_129_length_50193_cov_15.753879_56	KQKAKQCNVCKGNGKIRKVRKDGTPYARLNNCITCNSSGAGL
Station124_SUR_ALL_assembly_NODE_175_length_49109_cov_24.500734_17	KQKAMQCNVCKGNGKIRKVKKDGTPYAKPNNCATCNASGAGL
Station180_SUR_ALL_assembly_NODE_1460_length_53452_cov_8540064_16	RQKAKQCTTCSGTGQVRKVKKDGTSYARTSRCSSCDNTGGGL
CRP-345	KQKAVQCNACNGTGQIRKTRKDGTPYARTNKCTECDARGAGL
Station122_SUR_ALL_assembly_NODE_181_length_46530_cov_2366455_53	KQKAKQCQSCSGTGYTYKTRKDGTRYAKPNKCISCGATGAGL
Station100_DCM_ALL_assembly_NODE_115_length_56772_cov_11.658956_61	KQKAKQCQSCGGTGYTYKIRKDGTRYAKPNKCISCNATGAGL
CRP-1	KQKAKQCNDCNGVGKVYKTKKDGTQYARPNKCNGCDGVGAGL
Station209_MES_ALL_assembly_NODE_1330_length_49909_cov_40.446504_53	KQKAKQCRDCNGTGQIRKVRKDGTPYTRTNRCTSCDSTGAGL
Station155_DCM_ALL_assembly_NODE_846_length_55300_cov_83.906688_20	KTKAKQCGNCKGKGYYTRVKKDGNPFAKPSRCHECDTQGAGL
Station32_SUR_ALL_assembly_NODE_168_length_44742_cov_34.929196_37	KTKAKQCKTCFGKGKIRKVRKDGRLFANDNKCKICSGIGAGL
VTLG01010413_59	KTKAVKCIDCNGEGYIRKVKKDGNLYSKPSRCISCGTHGAGL
AFVG_25M368_21	KTKAEQCGYCKGTGYIRKIKKDGSPYAKPSVCPICDGNGAGL
Station56_MES_COMBINED_FINAL_NODE_696_length_45979_cov_7.928033_57	KTKAEKCWNCYGAGTIKKYKKDGSLYAKLPKCPKCNGEGAGF
Station111_SUR_ALL_assembly_NODE_80_length_53678_cov_74.079798_26	KTKAEKCWNCYGAGTIKKYKKDGSLYAKLPKCPKCNGEGAGF
Station30_DCM_ALL_assembly_NODE_173_length_48672_cov_19.356830_47	KTKAEKCHTCGGSGLIHKVKKDGTPYARLPKCAVCSGNGAGF
Station102_SUR_SMARTER_KIT_NODE_13_length_57979_cov_32.405255_25	KTKAEKCYTCGGSGLIHKVKKDGTPYARLPKCAECSGNGAGF
AFVG_25M29_62	KTVAERCPVCHGTGRIKKLKKDGTPYANLPKCVNCHALGAGF
Station124_SUR_ALL_assembly_NODE_149_length_53200_cov_7.343965_60	KTRAIKCRVCNGTGLMKKLKKDGTPYARPPKCPSCNGNGAGF
Station109_DCM_ALL_assembly_NODE_63_length_54399_cov_65.299573_17	KTIATKCTLCYGSGFIKKIKKDGTPYKNNTKCSNCDGSGAGL
VTLG01012261_1	KTVAVKCQACFGLGTQRKVRKDGKLYVKQPKCITCNGSGAGL
Station72_DCM_ALL_assembly_NODE_96_length_53747_cov_6.088970_43	KTQAVMCKTCNGTGKIRKTKKDGTPFARSNRCKVCNEAGAGL
Station125_SUR_ALL_assembly_NODE_176_length_53844_cov_13.480451_55	KTYASQCSTCKGTGKIWRTKKDGTPFARPNICKICDGAGAGL
Station67_SUR_ALL_assembly_NODE_218_length_50168_cov_41.016323_61	KTVAKQCTTCNGTGKTWKTKKDGTKYAKPNHCKTCDSQGAGL
VTLG01009300_11	KTKAGQCFACYGTGKQKKLKKDGTPYAKLPTCKECAGLGAGL
VTLG01001735_49	KTKAGQCFACYGTGKQKKLKKDGTPYAKLPTCKECAGLGAGL
VTLG01016337_2	KTKAEQCLDCLGKGYIHKTKKDGKPFAKQTKCKTCDTQGAGL
VTLG01001737_20	KTRAVKCSECNGSGNVYRTKKNGEPYAKPNKCPHCAGTGAGL
Station100_SUR_SMARTER_KIT_NODE_37_length_47254_cov_37.652196_19	KTVAMRCPDCNGSGSIRKIKKNGQPFARPTKCGTCNARGAGF
HMO-2011	KTIANQCATCNGTGQIRKTKKDGSPFANTNKCPACHGDGAGL
VTLG01014501_10	KTKAVQCTDCSGSGYITKTKKDGTPYARPNRCPTCNTAGAGL
VTLG01003473_28	KTKAVQCTDCSGSGYITKTKKDGTPYARPNRCPTCNTAGAGL
VTLG01016226_14	KTKAVQCTDCSGSGYITKTKKDGTPYARPNRCPTCNTAGAGL
Station122_SUR_ALL_assembly_NODE_166_length_49405_cov_6.331469_54	KTKAVQCTDCSGSGYITKTKKDGTPYARRNRCTTCNTAGAGL
Station52_SUR_ALL_assembly_NODE_69_length_51145_cov_20.899393_3	KTKATQCSVCKGTGQVRKVKKDGTLFARSNRCVSCNGNGAGL
VTLG01015678_48	KTKATQCGVCKGTGQIRKVKKDGTLFARHNRCTSCMGNGAGL
Station109_DCM_ALL_assembly_NODE_214_length_36053_cov_52.424246_31	KTSAEQCVTCKGTGYIRKTKKNGMPFARDSRCPHCDGAGAGL
VTLG01012193_31	KTKATQCRECNGSGQVRKVKKDGTPFARTNKCASCSGAGAGL
Station109_DCM_ALL_assembly_NODE_44_length_57835_cov_17164313_53	KTRATQCKTCNGTGYIRKVKKNGEPFAKPNRCPTCDTAGAGL
Station137_MES_DO_NOT_POOL_NODE_1277_length_39768_cov_5.796087_16	KTRAMQCKPCNGTGYIRKVKKNGEPFAKPNRCTDCDAAGAGL

CXXCXGX motif CXXCXGX motif

Station175_SUR_ALL_assembly_NODE_1109_length_54553_cov_45.340196_64	RTTASQCKVCRGVGRNKFAKKDGTMSKAVRICKECEGKGYLY
Station84_SUR_COMBINED_FINAL_NODE_786_length_46866_cov_5.412745_20	KTMAVRCNDCGGVGSTQKYKKTGDPYKTRSKCPTCEGLGAVY
Station168_DCM_ALL_assembly_NODE_1332_length_51622_cov_8.098222_36	RTNAVCCDACDGRAYIQKYKQQGEPYKNLSKCPSCAGVGAFY
AFVG_250M767_24	KTTASQCESCKGKGVIHKIKVDGTPYKKYTKCAVCDGDGYIY
Station64_SUR_ALL_assembly_NODE_290_length_52099_cov_9.196584_34	RTFAVHCTACDGTGKIFKTRKDGTKYKRGQTCAECGGVGTRY
Station123_MXL_ALL_assembly_NODE_578_length_34520_cov_6.319571_23	KTTASVCKTCKGTGKLRKIKKDGTPFSRAHTCHECGGAGMRY
Station56_SUR_ALL_assembly_NODE_197_length_43023_cov_20.478449_29	KTTASVCKTCKGTGKVRKIKKDGTPFSRAHTCHECGGAGMRY
Station32_SUR_ALL_assembly_NODE_173_length_44461_cov_6.338783_30	KTTASVCKTCKGTGKVRKIKKDGTPFSRAHTCHECGGAGMKY
Station78_MES_COMBINED_FINAL_NODE_767_length_50572_cov_18.758260_15	KTQVFQCTLCQGRGYSFPKKKDGSVGKAKRRCTKCDTAGVIF
Station76_MES_COMBINED_FINAL_NODE_839_length_55748_cov_7.182249_54	KTQVFQCTLCQGRGYSFPKKKDGSVGKAKRRCTKCDTAGVIF
Station78_MES_DO_NOT_POOL_NODE_755_length_45413_cov_13.032387_15	KTQVFQCTLCQGRGYSFPKKKDGSVGKAKRRCTKCDTAGVIF
Station76_SUR_ALL_assembly_NODE_229_length_55542_cov_4.284517_27	KTRAIQCKECLGVGYTLARKKDGTVGKAKRICPVCNKAGIVY
Station34_DCM_ALL_assembly_NODE_137_length_54335_cov_9.487012_49	KTKAIQCSDCFGKGFNLVRKKDGTIGKAKRLCKVCDAKGILY
Station76_SUR_ALL_assembly_NODE_247_length_52175_cov_12.583826_52	KTTAVSCADCQGKGYNLYVKKDGTVGKAKRICRTCNHKGIVY
Station109_DCM_ALL_assembly_NODE_73_length_52610_cov_15.844144_10	KTKASTCEICKGAKFVYKVRKDGTKFKKPNKCKNCDALGYTL
VTLG01015670.1_18	RTRAKTCSVCKGKGKVHKTKKDGSLFSKPNKCKECEARGYLL
Station125_MXL_ALL_assembly_NODE_201_length_52239_cov_17.971006_48	KTRAEQCNSCKGKGSYFKKKKTGGLYKKPTKCSVCNGTGFIY
Station124_MXL_ALL_assembly_NODE_149_length_53138_cov_21.503852_66	KTRAEQCNSCKGKGSYFKKKKTGGLYKKPTKCSVCNGTGFIY
Station123_SUR_ALL_assembly_NODE_131_length_52954_cov_6.687499_14	KTRAEQCNSCKGKGSYFKKKKTGGLYKKPTKCSVCNGTGFIY
Station123_MXL_ALL_assembly_NODE_163_length_53139_cov_53.603364_14	KTRAEQCNSCKGKGSYFKKKKTGGLYKKPTKCSVCNGTGFIY
Station122_DCM_ALL_assembly_NODE_181_length_53299_cov_30.317162_67	KTRAEQCNSCKGKGSYFKKKKTGGLYKKPTKCSVCNGTGFIY
Station155_DCM_ALL_assembly_NODE_963_length_51446_cov_150.706914_24	KTKAVQCSVCKGKGYVYKKKKDGSPYKKPSKCGACLG-GFNY
Station30_DCM_ALL_assembly_NODE_171_length_49093_cov_22.523003_43	KTKAEQCSSCKGKGYYYKYKKDGSPFKKSTKCITCNGLGYVF
Station82_SUR_COMBINED_FINAL_NODE_679_length_38824_cov_5.106528_10	KTKAMQCKKCKGKSFIYKIKKDGSQYKNKTKCATCNGQGYLY
Station102_DCM_ALL_assembly_NODE_213_length_48636_cov_5.295671_57	KKKAIQCSTCRGSGKTYKTKKNGERWAKPTKCPACAGEGYLY
Station175_ZZZ_ALL_assembly_NODE_735_length_49654_cov_98.119619_35	KKKAIQCSTCRGSGKTYKTKKNGERWAKPTKCPACVGEGYLY
Station155_DCM_ALL_assembly_NODE_929_length_52434_cov_43.564291_23	KKKAKQCKACYGTGKVRKMKKNGQPFAKPSKCPTCDAEGYLY
Station180_ZZZ_ALL_assembly_NODE_837_length_52361_cov_14.843345_18	KKKAKQCGVCYGKGKSYKTKKNGNPFARPTKCQGCEGEGYLY
Station201_DCM_ALL_assembly_NODE_752_length_43608_cov_16.579937_33	KTSARKCNTCNGTGYIRKTKKNGTPYSKDNKCSVCYAEGFIY
Station188_SUR_ALL_assembly_NODE_821_length_53046_cov_10.834783_50	KSHVKRCADCYGSGKLRKEKKDGTPYSKQSKCNSCGGNGYHV
Station25_DCM_ALL_assembly_NODE_326_length_46174_cov_23.453761_56	KTEARKCNVCHGKGQITKTKKDGKPYAKPTRCATCEGYGYLF
Station23_DCM_ALL_assembly_NODE_49_length_53806_cov_10.834254_46	KTNAVQCKTCYGSGKIRKIKKDGSPFAKESRCISCNAQGYLF
Station137_DCM_ALL_assembly_NODE_35_length_57516_cov_6.680740_34	KQRAVKCKTCYGTGYVRKTKKDGTPFAKPNRCVLCDAVGYQF
VTLG01002176.1_47	KQKAERCYECSGTGQIRKIRKNGTPHKNTNKCPVCSASGFLY
VTLG01013961.1_25	KQKAERCYECSGTGQIRKIRKNGTPHKNTNKCPVCSASGFLY
Station100_DCM_ALL_assembly_NODE_185_length_48277_cov_5.202999_26	KTKAVQCFTCYGTGKEKKIKKDGTPYIKQPSCKSCGGLGYHF
Station68_MES_COMBINED_FINAL_NODE_589_length_54561_cov_13.771566_17	KTIAVRCNNCYGSGIIRKIKKDGTPYINHPKCSTCNGSGYIF
Station102_SUR_SMARTER_KIT_NODE_15_length_55983_cov_15.559308_33	KTKAEQCKECKGFGKVRKVKKDGTPYARDNGCKTCNSLGFTL
Station125_MXL_ALL_assembly_NODE_184_length_53506_cov_3.844493_55	KTKANQCRECNGSGQIRKVKKNGVPFANTNKCTHCSGAGYTL

Supplementary Fig. 3 The alignment of the partial sequence of the DnaJ central domain from all HMO-2011-type isolates and MVGs. Two CXXCXGXG motifs are boxed. Conserved residues in the two CXXCXGXG motifs are shown in red. Other conserved residues are shown in bold.
